# Risky use of alcohol, drugs and cigarettes in a psychosis unit: a 1 1/2 year follow-up of stability and changes after initial screening

**DOI:** 10.1186/1471-244X-7-31

**Published:** 2007-07-06

**Authors:** Gunilla Cruce, Agneta Öjehagen

**Affiliations:** 1 Gunilla Cruce, Doctoral student Department of Clinical Sciences, Lund – Psychiatry and the Vardal Institute, Kioskgatan 19, Lund University Hospital, S-221 85 Lund, Sweden; 2 Agneta Öjehagen, Professor Department of Clinical Sciences, Lund – Psychiatry, Kioskgatan 19, Lund University Hospital, S-221 85 Lund, Sweden

## Abstract

**Background:**

Co-morbidity with substance use disorders negatively influences overall functioning in patients with psychosis. However, frequencies and courses of risky use of alcohol, drugs and cigarettes are rarely investigated in patients at psychosis units.

The purpose of this study is to describe the use of alcohol, drugs and cigarettes in patients at a psychosis unit over a 1 1/2 year period after them having taken part in a screening investigation including a feed-back of the results to personnel. Relationships with sex and age are also described.

**Methods:**

The patients' use of the substances was examined at baseline and at follow-up using three self-reporting instruments: Alcohol Use Disorders Identification Test (AUDIT), Drug Use Disorders Identification Test (DUDIT) and Fagerstrom Test for Nicotine Dependence (FTND).

**Results:**

One hundred and eighty-six patients out of 238 at baseline (78 percent) took part in the follow-up. Total AUDIT score decreased in women. Older men more often developed a risky alcohol use. Older women tended to reduce their risky drug habits. On a group level the habits mostly were stable, but 11 percent changed their alcohol habits and 15 percent changed their smoking habits from risky to no/low risky use, or vice versa. Nine percent changed their drug habits, predominantly from risky to no/low risky use.

**Conclusion:**

A more active approach towards alcohol, drug and smoking habits in psychosis units would probably be beneficial.

## Background

Co-morbidity with substance use disorders (SUD) is a common problem in patients with psychotic disorders. It is estimated that every other person with schizophrenia at some time also suffers from a SUD [1]. Misuse of alcohol, drugs and nicotine worsens the course of schizophrenia and other severe mental disorders with negative medical and social consequences [2-5]. Identification of hazardous use and intervention to prevent a negative course and a development of SUD could probably prove beneficial, with reduced risks for negative health and social consequences.

Most studies in this field concern substance misuse or substance dependence rather than hazardous use. A one year prospective study from the Epidemiologic Catchment Area study [6] found that the prevalence of SUD in persons with schizophrenia remained constant on a group level, while the course of SUD for individuals was varying. The subjects were categorized into four groups: those who remained abstinent, those who developed a SUD, those whose SUD had remitted and those whose SUD persisted over the study period.

In this paper the concept of "risky use" involves both a pattern of hazardous substance use referring to the *risk *of developing a harmful use and dependence, as well as having substance use disorders. This definition corresponds to cut off levels in the three questionnaires used, presented below.

A few studies carried out in psychiatric treatment settings have shown that screening procedures facilitate detection of risky use [7-9]. So far, studies on the identification of risky use, as well as on interventions to reduce risky use in patients with psychosis are rare. Hulse & Tait [10] found that hazardous or harmful alcohol use in inpatients with psychiatric disorders could be reduced by way of brief interventions. However, most of the patients in that study did not suffer from psychosis.

This paper focuses on the course of *risky alcohol, drug and smoking habits *1 1/2 years after a screening investigation including a feed-back of the results to the personnel. At baseline we found that one quarter of the sample had hazardous or harmful alcohol habits or drug-related problems or both. Eighteen percent had a hazardous or harmful alcohol use, including seven percent who reported alcohol habits indicating heavy abuse or dependence. Nine percent reported drug-related problems, women more often than men (14 percent versus 5 percent). Forty-nine percent were smokers. Half of the smokers reported questionnaire scores indicating a strong or extreme dependence. Multivariate analyses showed that female sex and being a smoker were independently related to an increased risk of having drug-related problems [11].

The primary aim of the present study is to analyze stability and change in alcohol, drug and smoking habits among patients in a psychosis unit. A secondary aim is to investigate whether sex and age are associated with patterns of stability or change.

## Methods

### Participants and setting

Two hundred and forty-one patients who had taken part in a study of the prevalence of risky use and misuse of alcohol, drugs and cigarettes, were asked to participate in a follow-up study one and a half years later. The sample consisted of 18 inpatients and 223 outpatients at the Psychosis Unit at the Department of Psychiatry Lund University Hospital in Sweden. Patients in the inpatient sample were more often nicotine dependent (p = 0.037) and tended more often to have risky alcohol habits (p = 0.063) compared to the outpatient sample. Fifty-seven percent of the total sample were male; the mean age was 45 years (SD 12 years) and the median age 44 years (range 21–79 years). The mean age for women was greater than that for men, 49 years (SD 12) versus 43 years (SD 12), p < 0.001. Most of the patients had a diagnosis of schizophrenia (70 percent). The remaining patients suffered from persistent delusional disorders, acute and transient psychotic disorders, schizoaffective disorders and psychosis NOS.

### Procedures

The screening results at baseline had been reported in writing to the psychiatrist in charge of each patient. The former was then meant to communicate the results to the patient and key-worker, and if needed carry out an intervention. Unfortunately, we have no information *if *and in which case *how *the patients received this information. No support was asked for on how to provide feedback and perform interventions for the patients and we thus had no opportunity to follow these procedures [11].

One and a half years after collecting of baseline data, the key-workers and psychiatrists were requested to ask the patients to take part in a follow-up screening. The same procedure as was applied in the first screening where the patients could answer the questionnaires together with the caregiver or by themselves was applied for the second screening. A written feedback was sent to the psychiatrist in charge of the patient to be forwarded to patients and key-workers.

The study was approved by the Lund University Medical Ethics Committee, LU 763-03 and informed consent was obtained from the participants.

### Instruments

The patients reported their alcohol, drug and cigarette habits at follow-up using the same three self-reporting questionnaires (AUDIT, DUDIT and FTND) as were used at baseline. The Alcohol Use Disorders Identification Test (AUDIT) has been used to identify hazardous or harmful use in primary care [12], and it has also been used successfully among persons with schizophrenia [8]. It consists of 10 items, and the maximum score is 40. A recommended cut-off score, ≥ 6 for women and ≥ 8 for men indicates a hazardous or harmful alcohol use, as well as a possible dependence [13]. Women are recommended a lower daily intake due to higher sensitivity to the acute and chronic effects of alcohol and therefore the female cut-off score is 25 percent lower. In this presentation persons who score 0 are defined as "abstainers". Women who score 1 – 5 are defined as "non-hazardous alcohol users", as are men who score 1 – 7. Those scoring above cut off are labelled "hazardous or harmful users". Scores 13–18 in women and 15–18 in men indicate a "heavy abuse", according to Bergman and Källmén [14]. Scores 19 or more indicate "alcohol-related problems including dependence" [13]. In this paper, abstainers and patients with habits indicating a non-hazardous use are called "no/low risky users" and persons with habits indicating a hazardous use, heavy abuse and dependence are called "risky users".

The Drug Use Disorders Identification Test, DUDIT [15], with a maximum score of 44, comprises 11 questions that correspond to the items of the AUDIT. In a study from the general Swedish population the preliminary recommendation is a cut-off score of ≥ 2 for women and ≥ 6 for men to identify persons with drug-related problems. These cut off scores emanate from T-scores at 2 SD from the mean in that sample [15]. Following the recommendation we define women who score 1 as well as men who score 1–5 as "non-hazardous drug users". A corresponding categorization to that used for alcohol use is also applied here, where patients with different drug use habits are categorized into "no/low risky users" (drug-free and scores indicating a non-hazardous drug use) and "risky users" (scores indicating drug-related problems).

The Fagerstrom Test for Nicotine Dependence, FTND [16] includes six questions about daily cigarette consumption and measures the extent of cigarette smoking and smoking behaviour. Test subjects are categorized in the following way: "very low dependence" (0–1 points), "some dependence" (2–3 points), "dependence" (4–5 points), "strong dependence" (6–7 points), and "extreme dependence" (8–10 points), respectively.

Scores on the three questionnaires cannot directly be converted into diagnostic categories. In order to establish a diagnosis of substance use disorder a clinical investigation is needed.

### Statistics

Data analysis was performed using SPSS for Windows 12.0.1. Proportions were compared by use of the Chi-square test, and comparisons between scores with non-parametric tests (Mann-Whitney U-test). When appropriate, Fisher's Exact Test was used. Wilcoxon matched pairs test was used to investigate differences in repeated measurements.

## Results

### Follow-up sample

At follow-up three patients, one woman and two men had died. None of them had had a hazardous or harmful alcohol use, one man and one woman had been moderate smokers and the woman had had drug-related problems.

The time between the first and the second screening varied with a mean follow-up period of 18 months (SD 4) and a median of 18 months (range 13 months – 26 months). One hundred and eighty-six patients out of 238 at baseline (78 percent) took part in the follow-up, 83 out of 103 women (81 percent) and 103 out of 135 men (76 percent). The follow-up sample consisted of 45 percent women and 55 percent men. In the baseline study the sample was divided into two age groups, those 44 years or younger and those 45 years or older, a cut-off based on median age at baseline. Seventy-eight percent from both the younger group and from the older group participated in the follow-up.

Baseline total AUDIT and DUDIT scores did not differ between those who dropped out and those followed up, nor did the proportions scoring above cut-off on these instruments. Neither total FTND scores nor the proportions of smokers differed between drop outs and those followed up. However, female patients who were followed up were more often smokers at baseline than were female drop-outs (44/83, 53 percent versus 5/21, 24 percent, p = 0.03).

We have no specific or quantified information about why the majority of those who did not take part in the follow-up chose not to do this. Some had completed their treatment at the psychosis unit; others had declined to take part or had not been asked to participate because of inconvenience.

### Alcohol use

The proportions of patients with abstinence, non-hazardous alcohol use, hazardous or harmful alcohol use, heavy abuse or dependence did not differ between baseline and follow-up (table 1). The average score decreased, mainly due to decreased scores in female patients.

**Table 1:** Proportions of patients in AUDIT levels, and mean scores at baseline (I) and follow-up (II).

		All	Women	Men	Age ≤ 44	Age > 44
		n = 178	n = 79	n = 99	n = 91	n = 87

		I – II	I – II	I – II	I – II	I – II

Total abstinence (0 p)		28%–35%	34%–46%	22%–26%	30%–33%	26%–36%
Non-hazardous use (♀ 1–5 p; ♂ 1–7 p)		53%–49%	54%–47%	52%–50%	46%–48%	60%–49%
Hazardous or harmful use (♀ ≥ 6 p; ♂ ≥ 8 p)		19%–17%	11%–8%	25%–24%	24%–19%	14%–15%
- (hazardous use)		(11%–11%)	(4%–3%)	(16%–18%)	(12%–11%)	(8%–12%)
- (heavy abuse)		(3%–3%)	(4%–4%)	(3%–3%)	(3%–6%)	(3%–1%)
- (dependence)		(5%–2%)	(4%–1%)	(6%–3%)	(8%–2%)	(2%–2%)
						
*Total score*	*I*	*4.1 ± 6.0*	*3.0 ± 5.5*	*5.1 ± 6.3*	*5.0 ± 6.9*	*3.3 ± 4.9*
*(0–40 points)*	*II*	*3.4 ± 5.3 *^*a*^	*2.3 ± 4.9 *^*b*^	*4.3 ± 5.5*	*3.8 ± 5.2*	*3.0 ± 5.4*

a) In total, all scored higher in screening I than in screening II, p = 0.05.

b) Women scored higher in screening I than in screening II, p = 0.03.

As presented in table 2 the individual courses varied, and four *patterns of change *were identified within the sample:

**Table 2:** Patterns of change in alcohol, drug and cigarette use among persons with psychosis at 1 1/2 years follow-up.

	**Status at follow-up**									
**Status at baseline**	No/low risky use				Risky use					
**Alcohol use**	Abstinence		Non- hazardous use		Hazardous/harmful use		Heavy abuse		Dependence	

No/low risky use	*n*	*%*	*n*	*%*	*n*	*%*	*n*	*%*	*n*	*%*
Abstinence, n = 49	38	78	11	22	0	0	0	0	0	0
Non-hazardous use, n = 95	19	20	68	72	7	7	1	1	0	0
Risky use										
Hazardous use, n = 19	0	0	7	37	10	53	2	11	0	0
Heavy abuse, n = 6	1	17	0	0	1	17	1	17	3	50
Dependence, n = 9	2	22	2	22	2	22	2	22	1	11

**Drug use**	Abstinence		Non- hazardous use		Drug-related problems					

	*n*	*%*	*n*	*%*	*n*	*%*				
Abstinence, n = 149	141	95	6	4	2	1				
Non-hazardous use, n = 4	2	50	1	25	1	25				
Drug-related problems, n = 17	12	71	0	0	5	29				

**Cigarette use**	Non-smoking				Smoking					

	*n*	*%*			*n*	*%*				
Non-smoking, n = 92	77	84			15	16				
Smoking, n = 94	13	14			81	86				

A. Those who remained no/low risky alcohol users (no/low risky use → no/low risky use).

B. Those who developed a risky alcohol use (no/low risky use → risky use).

C. Those who restored a no/low risky alcohol use (risky use → no/low risky use).

D. Those who remained risky alcohol users (risky use → risky use).

*Pattern A *(no/low risky use → no/low risky use). Among no/low risky users (including both abstainers and non-hazardous users) 94 percent (n = 136) remained no/low risky users between the two measurements. Older women more often remained no/low risky users than older men (100 percent versus 88 percent, p = 0.04). Within the subgroup of total abstainers 78 percent continued to abstain at follow-up, with women doing so more often than men (89 percent versus 64 percent, p = 0.046) while the rest continued a no/low risky use.

*Pattern B *(no/low risky use → risky use). All eight patients (6 percent) who developed a risky use had been using alcohol at baseline. Older men developed a risky use more often than older women (12 percent versus 0 percent, p = 0.04).

*Pattern C *(risky use → no/low risky use). Thirty-five percent (n = 12), representing all three subgroups of risky use (including hazardous or harmful use, heavy abuse and dependence) achieved a no/low risky use level and no differences between sexes or age groups were found. Four of the nine patients with scores indicating dependence at baseline reported no/low risky use at follow-up. The mean score for these nine patients decreased from 24 points (SD 2.0) to 9 points (SD 7.5), p = 0.008.

*Pattern D *(risky use → risky use). Among those with a risky use at baseline, 65 percent continued to be risky users at follow-up (n = 22), with no differences between sexes or age groups. The total scores did not change in this group, but for 23 percent (5/22) alcohol-related problems deteriorated, i.e. they moved from one subgroup to another within the category of risky use. On the other hand a similar proportion improved their habits, although remaining risky users at follow-up.

### Drug use

The proportion of subjects reporting drug-related problems was reduced by almost 50 percent between baseline and follow up (table 3), eight out of seventeen.

**Table 3:** Proportions of patients in DUDIT levels, and mean scores at baseline (I) and follow-up (II).

		All	Women	Men	Age ≤ 44	Age > 44
		n = 170	n = 76	n = 94	n = 88	n = 82

		I – II	I – II	I – II	I – II	I – II

No drug use (0 p)		88%–91%	86%–96%	89%–87%	90%–90%	85%–93%
Non-hazardous drug use (♀ 1 p; ♂ 1–5 p)		2%–4%	0%–0%	4%–7%	2%–4%	2%–4%
Drug-related problems (♀ ≥ 2 p; ♂ ≥ 6 p)		10%–5% ^c^	15%–4%	6%–5%	8%–6%	12%–4%
						
*Total score*	*I*	*1.2 ± 4.7*	*1.0 ± 3.4*	*1.3 ± 5.5*	*1.1 ± 5.1*	*1.3 ± 4.1*
*(0 – 44 points)*	*II*	*0.9 ± 4.3*	*0.3 ± 1.6*^*d*^	*1.4 ± 5.5*	*1.1 ± 4.7*	*0.8 ± 3.7*

c) In total, the proportion of drug-related problems decreased between baseline and follow-up, p < 0.001.

d) Women scored higher at baseline than at follow-up, p = 0.016

The number of women with drug-related problems decreased, especially among the older women, where six out of seven had turned to a no/low risky level. Women also reduced their total scores at follow-up (table 3). This reduction was, however, statistically significant in older women only, 1.1 (SD 3.2) versus 0.2 (SD 1.4), p = 0.017 *(not tabulated)*.

The patterns of change in drug use will be presented using the same categories as in the changes in alcohol use (see above):

*Pattern A *(no/low risky use → no/low risky use). As shown in table 2 the majority of those who abstained or were non-hazardous users at baseline (n = 153) remained no/low risky users at follow-up (98 percent). However, five percent (8/149) started to use drugs during the investigation period.

*Pattern B *(no/low risky use → risky use). Only three patients out of 153 (two percent) developed a risky use, two of whom were abstainers at baseline.

*Pattern C *(risky use → no/low risky use). The largest change occurred in the group of patients with a risky drug use at baseline: Twelve out of seventeen changed their drug habits from a risky to a no/low risky level (abstinence in fact).

*Pattern D *(risky use → risky use). Twenty-nine percent (5/17) remained as risky users.

There were no differences between sexes or age groups in any of these drug use patterns.

### Cigarette-smoking

The proportion of smokers did not differ between baseline and follow-up; neither did proportions at any FTND level (table 4). No differences in FTND scores between baseline and follow-up were found.

**Table 4:** Proportions of patients in FTND levels, and mean scores at baseline (I) and follow-up (II).

		All	Women	Men	Age ≤ 44	Age > 44
		n = 186	n = 83	n = 103	n = 94	n = 82

		I – II	I – II	I – II	I – II	I – II

Non-smokers		50%–48%	47%–45%	52%–52%	50%–51%	49%–46%
Very low dependence (0–1 p)		7%–10%	6%–7%	8%–13%	10%–11%	4%–10%
Some dependence (2–3 p)		5%–3%	5%–4%	6%–3%	3%–1%	8%–5%
Dependence (4–5 p)		11%– 11%	12%–12%	11%–10%	9%–6%	14%–15%
Strong dependence (6–7 p)		13%–14%	13%–16%	13%–13%	14%–18%	12%–10%
Extreme dependence (8–10 p)		14%–13%	17%–17%	12%–11%	15%–13%	13%–14%
						
*Total score*	*I*	*5.7 ± 2.4*	*6.0 ± 2.4*	*1.3 ± 5.5*	*6.0 ± 2.4*	*5.5 ± 2.4*
*(0 – 10 points)*	*II*	*6.0 ± 2.4*	*6.2 ± 2.5*	*1.4 ± 5.5*	*6.2 ± 2.3*	*5.9 ± 2.6*

Approximately the same proportion that quitted smoking, started to smoke (table 4).

## Discussion

The proportions of subjects with habits indicating risky *alcohol *and *nicotine *habits were approximately equal at baseline and at follow-up, mainly due to stable habits, but also because of similar proportions of patients changing from risky habits to no/low risky use, and vice versa. Eleven percent of the patients changed alcohol habits and fifteen percent either had begun to smoke or had quitted smoking. Regarding differences between sexes or age groups the only differences found were in women, whose AUDIT score had decreased, and in older men, who more often developed a risky alcohol use.

Risky *drug *habits had decreased markedly between baseline and follow-up, mainly due to changes among the older women. In most patients drug habits were stable, but nine percent of the sample changed their drug habits, predominantly from risky to no/low risky use.

These results suggest that the prevalence of risky alcohol and smoking habits (but not risky drug habits) in Swedish patients treated at a psychosis unit persists over a period of one to two years.

A lower prevalence of risky use and misuse is found in the present study in comparison with earlier research [7,9,17,18]. Maybe selection bias in the original population (frequencies of risky alcohol and drug use may have been higher among those who did not take part in the study), as well as the non-anonymous interview procedure have had a restraining effect on the reported frequencies. Furthermore it is also possible that a difference in settings and the relatively low rates of risky alcohol use in the Swedish general population, might explain some of these differences. Even though we found a lower prevalence than has been found in earlier studies from Sweden and other countries, it can still be stated that too many patients in psychosis units use alcohol, drugs and cigarettes in a risky way.

The use of population-based risk cut offs for this sample may result in an under-detection of risk, given the potential sensitivity of the group to very small amounts of substances [19]. This assumption is consistent with findings from the National Survey of Mental Health and Well-Being in Australia: persons with psychosis who reported cannabis use were almost three times more likely to be dependent users than persons without psychosis who had used cannabis. Furthermore, persons with psychosis who had used alcohol were five times more likely to be alcohol dependent than persons without psychosis who had used alcohol. The authors conclude "It may be that such persons are at higher risk of developing problematical use when they use these drugs" [20]. According to these results, a lower cut-off for *risky use *perhaps should be used among persons with severe mental disorders.

Unfortunately, we do not know to what extent and how the feed-back at baseline was communicated to the patients. Whether or not changes could be explained by the screening itself *or *to feed-back of results *or *to feed-back together with some type of further intervention are questions which cannot be answered by the present study.

The screening procedure in itself might influence the course by way of self-reflection and a self-monitoring process. A positive effect of screening only, as well as screening combined with an intervention in a sample with hazardous or harmful alcohol habits was found in a study in primary care by the WHO and Brief Intervention Study Group [21]. However, after nine months males had reduced their consumption after intervention more than those who had only been screened, while in females no difference was found between the intervention and the control groups.

Women in the present study were more likely than men to change risky alcohol and drug behaviours. This finding is consistent with other studies [22-24] reporting better treatment results in women with alcohol problems. However, a problem in the present sample is the low numbers of risky alcohol and drug habit levels, resulting in a probable risk of not detecting significant differences in change.

Despite the fact that the prevalence of risky *alcohol *and *smoking *habits was stable over time, the individual course of these habits was variable. We found, as Cuffel & Chase [6] had done, a relatively high level of individual flexibility within the sample, which in the present study meant that a similar proportion of patients were developing risky habits as were returning to no/low risky behaviours. The proportion of patients changing *drug *habits was quite similar to the proportions of patients changing alcohol and smoking habits, but most of these patients changed from risky to no/low risky drug habits.

The samples in the study by Cuffel & Chase [6] as well as in the study by Bartels and colleagues [25] mentioned below, included subjects with a SUD, and are thus not totally comparable to the present sample, which also includes subjects with habits indicating a *risky use*. Bartels and colleagues in their seven-year naturalistic follow-up study found that SUD in severely mentally ill persons tends to persist over many years. However, they stated that there is a need to make a distinction between *abuse *and *dependence *because they found that remission in alcohol and drug abusers is substantially more frequent than in those who are dependent. With these results in mind when looking at the data concerning the present sample of patients with mainly *hazardous *habits, one may speculate that we should have expected to have found more individuals changing from risky to no/low risky levels than was the case. The results in the present study are clearly not in line with Bartels and colleagues' findings and we do not know why the prevalence of hazardous habits in our sample did not decrease. It may be that people become worried about their substance use and change their habits only when they experience deterioration in health.

It is surprising to note that in contrast to the findings reported by Bartel and colleagues, the largest proportion (16/26 = 62%) of patients reporting a no/low risky level of use were patients with probable alcohol dependence (4/9) and drug-related problems (12/17) at baseline. This pattern may have been impacted by motivational interventions and further treatment by key-workers and psychiatrists in response to the feedback results.

The detection of risky use provides staff with an opportunity to actively prevent harmful consequences and dependency. Lack of skills and time are found to be impediments among staff for motivational interventions and treatment [26,27]. In the present study risky alcohol and nicotine habits did not change, which might indicate need for support for motivational intervention. Risky drug habits were, however, reduced by 50 percent which raise questions about staff attitudes and opinions on different substances. In Sweden, the *use *of illegal drugs, is a breach of the law and underscores the seriousness of risky drug use. It is likely that staff act more energetically to motivate risky users to quit their drug use, than to reduce their risky alcohol and cigarette use. Staff might also react more negatively if patients misuse legal drugs prescribed by their own psychiatrist. Staff (and patients) may overlook risky alcohol and cigarette use since they are not aware of the possible serious medical, psychological and social consequences of even small amounts of these substances. There is probably a need for further education and skills training in this area among staff in psychosis units.

## Conclusion

The results of the present study underscore the need of a more active approach to alcohol, drug and smoking habits within mental health services, including routine screening, a structured feed-back to patients, and motivational interventions directed to patients with psychotic disorders.

## Competing interests

The authors declare that they have no competing interests.

## Authors' contributions

GC participated in the design of the study, collected, analysed and interpreted the data, and drafted the manuscript. AÖ designed the study, was involved in analysing and interpreting the data and was helpful drafting the manuscript. Both authors approved the final manuscript.

## Pre-publication history

The pre-publication history for this paper can be accessed here:


